# *Escherichia coli* O157 Exposure in Wyoming and Seattle: Serologic Evidence of Rural Risk

**DOI:** 10.3201/eid0910.020254

**Published:** 2003-10

**Authors:** Jason P. Haack, Srdjan Jelacic, Thomas E. Besser, Edward Weinberger, Donald J. Kirk, Garry L. McKee, Shannon M. Harrison, Karl J. Musgrave, Gayle Miller, Thomas H. Price, Phillip I. Tarr

**Affiliations:** * University of Washington School of Medicine, Seattle, Washington; † Children’s Hospital and Regional Medical Center, Seattle, Washington; ‡ Washington State University, Pullman, Washington; § Star Valley Hospital, Afton, Wyoming; ¶ Wyoming Department of Health, Cheyenne, Wyoming; # Puget Sound Blood Center, Seattle, Washington

## Abstract

We tested the hypothesis that rural populations have increased exposure to *Escherichia coli* O157:H7. We measured circulating antibodies against the O157 lipopolysaccharide in rural Wyoming residents and in blood donors from Casper, Wyoming, and Seattle, Washington, by enzyme immunoassay (EIA). EIA readings were compared by analysis of variance and the least squares difference multiple comparison procedure. Rural Wyoming residents had higher antibody levels to O157 LPS than did Casper donors, who, in turn, had higher levels than did Seattle donors (respective least squares means: 0.356, 0.328, and 0.310; p<0.05, Seattle vs. Casper, p<0.001, rural Wyoming vs. either city). Lower age was significantly correlated with EIA scores; gender; and, in rural Wyoming, history of bloody diarrhea, town, duration of residence, and use of nontreated water at home were not significantly correlated. These data suggest that rural populations are more exposed to *E. coli* O157:H7 than urban populations.

*Escherichia coli* O157:H7 is an important human pathogen. This organism can affect humans in a variety of ways, ranging from asymptomatic carriage (*1*) to diarrhea, bloody diarrhea (the most common manifestation of illness in culture-proven cases), and the postdiarrheal thrombotic microangiopathy, hemolytic uremic syndrome (HUS) (*2*). Infections with *E. coli* O157:H7 in the Pacific Northwest of the United States have been endemic (*3*) and epidemic (*4*). Vehicles transmitting this pathogen include unpasteurized milk and juice (*5*,*6*), undercooked beef (*7*), drinking water (*8*), and contact with infected persons (*9*).

Data from the Centers for Disease Control and Prevention (CDC) demonstrate higher incidences of *E. coli* O157:H7 infections in rural counties in the United States than urban (Paul Mead, unpub. data). Worldwide, rural populations have been postulated to be at greater risk for exposure to *E. coli* O157:H7 by virtue of increased exposure to animals or their excreta in Scotland (*10*,*11*); dairy farm visits have been implicated as a source for infection in Finland (*12*) and the United States (*13*); and animal contacts are a risk factor for the development of HUS in Switzerland (*14*). Serologic studies from Canada demonstrated higher frequencies of antibodies to the O157 lipopolysaccharide (LPS) side chain among residents of rural areas compared to residents of urban areas (*15*), and in Wisconsin children, manure and sheep contact were recently demonstrated to be risk factors for O157 seropositivity (*16*). Taken together, these data suggest more intense or more frequent human exposure to *E. coli* O157:H7 in nonurban areas.

Populations in the Pacific Northwest and Rocky Mountain states provide an opportunity to assess the frequency of exposure to *E. coli* O157:H7 through serologic studies. Antibodies to the O157 LPS follow natural infection with *E. coli* O157:H7 (*17*) and are believed to be quite specific (*18*) because they are rarely found in healthy people. Thus, circulating antibodies to the O157 LPS are potential markers of population exposure to *E. coli* O157:H7. We therefore attempted to assess the distribution of antibodies to this antigen in three different populations, encompassing a gradient of population density.

## Methods

### Study Participants

Participants were selected for inclusion in this study if they were >16 years of age, weighed >54 kg, and participated in voluntary cholesterol screening in several rural western Wyoming towns (population A), or donated blood to the Wyoming State (population B) or Puget Sound (population C) blood banks, and provided informed consent. The Institutional Review Boards of the Children’s Hospital and Regional Medical Center (Seattle, Washington) and the University of Wyoming (Laramie, Wyoming) approved this study before participants were enrolled.

Population A consisted of 485 residents of Star Valley, Wyoming. This valley has extensive agricultural land usage and consists of a series of small towns along U.S. Highway 89 in Lincoln County in the northwestern part of the state; town populations range from 100 to 1,200 residents. One of these towns had an *E. coli* O157:H7 outbreak in 1998 (*19*). During a local health fair conducted in the towns of Afton, Thayne, and Alpine during May 1999, participants donated 5 mL of blood during phlebotomy for screening to detect hyperlipidemia and answered a questionnaire regarding age, gender, treated versus nontreated domestic water supply, history of bloody diarrhea, location of sampling, and town of residence.

Population B was composed of 196 blood donors at United Blood Services (UBS), Casper, Natrona County, Wyoming. UBS, Wyoming’s only blood bank, obtains most of its blood from donors residing within Casper, the second largest municipality in the state (population 49,644 residents [20] in 52.8 km^2^ [Mike Jun, pers. comm.]). This site was chosen for study because of its presumed intermediate intensity of exposure to agriculture. Volunteers provided 5 mL of whole blood in a separate tube as part of the donation. Gender and age data were available for 127 (65%) of the participants.

Population C consisted of 104 blood donors at the Puget Sound Blood Center, Seattle, Washington, all of whom resided in urban or suburban Puget Sound municipalities (including Seattle and surrounding communities) and provided an additional 5 mL of blood for research. This group was presumed to have less agricultural exposure than populations A or B. Age and gender were recorded for all but two donors. Serum specimens from populations B and C were collected during the spring and summer of 1999.

Cattle and human density data for Lincoln, Natrona, and King Counties are provided in Table 1. These data demonstrate the rural-to-urban human population density and cattle-to-human ratios for the populations chosen. Blood samples were centrifuged within 3 hours of donation; serum samples were separated from the packed erythrocytes and stored at –70°C until assayed.

**Table 1:** Cattle and human density data for Lincoln and Natrona Counties, Wyoming, and King County, Washington^a^ (states of donation)

Characteristics of regions	Population A (Lincoln County)^b^		Population B (Natrona County)^b^		Population C (King County)^b^		
Residents	14,573	66,533		1,737,034		
Cattle	53,000	62,000		30,500		
Cattle/km^2^ Cattle/resident	5.1 3.6	4.5 0.93		5.5 0.017		
Residents/km^2^	1.4	4.8		315.4		

^a^Source: reference *20*. Source: reference *21*.

### Detection of Antibodies to *E. coli* O157 LPS

*E. coli* O157:H7 LPS was purified from strain 86-24 (*21*) by using phenol extraction (*22*). Purified LPS underwent electrophoresis in a 12% polyacrylamide sodium dodecyl sulfate-polyacrylamide gel electrophoresis gel and then was transferred to a membrane and probed with antibodies to the LPS antigen (*23*). Serum specimens were screened for antibodies to the O157 LPS with an enzyme immunoassay (EIA) (*24*), modified by CDC. Briefly, in preliminary experiments, the optimal LPS concentrations for coating plates and diluting serum samples were determined by block titration with phosphate-buffered saline (PBS) (0.01 M, pH 7.2) and a known positive human sample. Optimal dilutions of 1:20 for serum and 1:160 of the antigen stock solution (corresponding to 200 μL of a resuspended pellet from a 50-mL overnight culture in Luria broth [*25*]) were demonstrated (data not shown).

Next, individual wells of an Immulon II plate (Dynex Technology, Franklin, MA) were coated with diluted antigen and incubated (4°C, overnight) to enable the target antigen to adhere to the plates. Then, 150 μL of PBS containing 1% fetal bovine serum and 0.5% nonfat dry milk (PBS-M-FBS) was placed in each well. The plates were then incubated in a blocking step (room temperature, 2 hours) to prevent nonspecific antibody adherence to the plates. Fluid was then removed, and the plates were washed four times with 0.01 M PBS containing 0.05% Tween 20 (PBS-T).

Ten microliters of diluted human serum sample was added to 190 μL of PBS-M-FBS containing 0.05% Tween 20 (PBS-T-M-FBS), placed into wells of the microtiter plates, and incubated (37°C, 1 h). The fluid was then removed, and the plates were washed four times with PBS-T. One hundred microliters of alkaline-phosphatase–labeled goat antihuman immunoglobulin (Ig) G/IgM/IgA (heavy and light chain) (Kierkegaard & Perry Laboratories, Inc., Gaithersburg, MD), diluted 1:10,000 in PBS-T-M-FBS, was then placed in each well and incubated (37°C, 1 h). The plates were then washed four times with PBS-T.

A substrate solution containing 97 mL diethanolamine, 0.2 g sodium azide, 100 mg magnesium chloride-6H_2_O, and 800 mL distilled water was adjusted to a pH of 9.8 with 1 M hydrochloric acid. One p-nitrophenyl phosphate tablet (Sigma Chemical Co., St. Louis, MO) was then added to 5 mL of substrate solution. One hundred microliters of this solution was placed in each well and incubated (room temperature, 25 min). The reaction was halted by adding 50 μL of 3 M sodium hydroxide to each well, and the optical densities of each well were read in a dual wavelength micro-EIA reader at λ = 405 nm with background correction at λ = 540 nm (Elx 800, Bio-Tek Instruments, Winooski, VT). Each serum sample was assayed in duplicate, and the values were averaged.

Serum from a patient with HUS caused by *E. coli* O157:H7 and serum from a study participant without known *E. coli* O157:H7 infection in population A were included as duplicates on each plate as positive and negative controls, respectively, and values were averaged. Each plate also contained controls without antigen or primary or secondary antibody. All plates were normalized linearly in relation to the positive control in the first group of serum samples tested.

### Analysis

The complete dataset was first studied by analysis of variance (ANOVA, Proc GLM, SAS Institute, Inc., Cary, NC) in a model with EIA readings as the dependent variable, gender and town/city as class-independent variables, and age as a continuous independent variable. Initially, all interactions were included in the model, but interactions not contributing significantly to the model were dropped from subsequent analyses. Multiple comparisons were analyzed by using the protected Fisher least squares differences (LSD) test after confirming that the p value of the model as a whole was <0.05. The data were approximately normally distributed, as demonstrated by a Wilk-Shapiro statistic >0.98 (either for the dataset as a whole or for each region separately, Proc UNIVARIATE, SAS Institute, Inc.) and by visualization of the residuals plot. However, as assumptions of normal distribution of the data are difficult to confirm robustly, the data were also analyzed after transformation of these values into binary form with arbitrarily chosen cutpoints at the 80th and 90th percentiles of the EIA scores or with the entire range of EIA scores categorized at 0.05 increments, using stepwise logistic regression (Proc LOGISTIC, SAS Institute, Inc.) with the same independent variables as described above for the ANOVA (*26*). Statistically, p values <0.05 were considered significant for comparisons, and p values <0.05 were set as the criterion for entry and for retention into logistic regression models.

## Results

We tested 785 serum samples for antibody reactivity. The summary statistics for the O157 EIA are provided in the Figure (panel A), and the demographic characteristics of each population contributing these serum specimens are provided in Table 1. The average age of the study participants was significantly younger in populations B and C, than in population A. The populations did not differ significantly with respect to gender.

**Figure:**
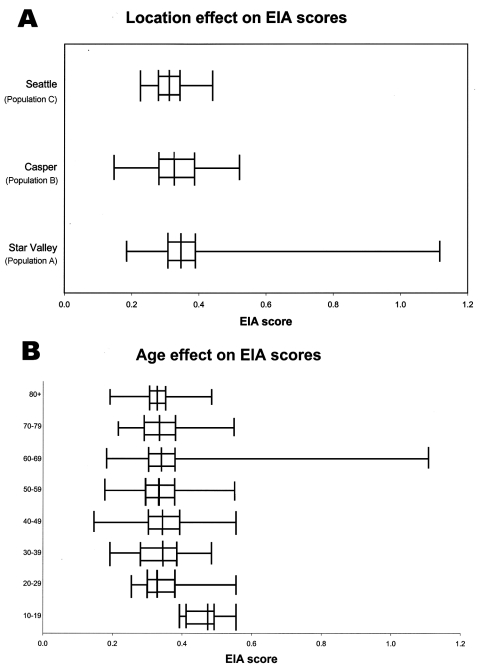
Box plot analysis for enzyme immunoassay (EIA) values, by populations. X axis represents EIA scores for study participants.Vertical line in each box represents the median for each population. The left and right borders of each box are the 25th and 75th percentiles of each population, respectively. The extensions beyond each box represent the lowest and highest values for each population. Panel A demonstrates the results for each population; panel B demonstrates the results by age.

EIA scores were significantly related to both age (p<0.01) and population (p<0.001) in ANOVA. The effect of age is illustrated in the Figure (panel B). Age and gender information were not available for 35% of population B, but the distributions of EIA scores did not differ between those observations with and without these data, suggesting that the loss of data did not bias the results. The same two variables, age and population, also were significantly (p<0.05) associated with EIA score in logistic regression analysis, irrespective of whether the dependent variable modeled was the 80th percentile (Table 2) or the 90th percentile of the EIA score or increased increment of the EIA score (data not shown).

**Table 2:** Age, gender, history EIA value distribution in each population study, and median and range of OD for the EIA readings^a^

	Population A	Population B	Population C
Characteristics of populations	N =485	N =196	N =104
Age (y) Mean (SD)	57 (15)	45 (12)	45 (12)
			
Male: female (not reported)	225:260	56:71 (69)	56:46 (2)
OD (EIA units) Least squares mean	0.356^b^	0.328^c^	0.310
Median (range)	0.348 (0.185–1.115)	0.328 (0.149–0.522)	0.312 (0.222–0.441)
EIA, 80th percentile N+ (%) EIA, 90th percentile N+ (%)	107 (22.0%) 60 (12.4%)	40 (20.4%) 19 (9.7%)	5 (4.8%) 2 (1.9%)

^a^EIA, enzyme immunoassay; OD, optical density; SD, standard deviation. ^b^p<0.001, population A (Star Valley) vs. either Casper or Seattle. ^c^p< 0.05, population C (Seattle) vs. population B (Casper).

Within the rural Wyoming group, the data allowed analysis of O157 LPS EIA values’ association with additional variables, including the duration of residence in the area, occurrence of bloody diarrhea, use of a chlorinated water supply at the residence, and town of residence within Star Valley (Table 3). None of these variables was significantly associated with EIA mean values. Residents living in the area for <2 years tended to have higher average EIA values (0.380) than those who resided there for longer periods (2–5 years, 0.346; >5 years, 0.355).

**Table 3:** Age, gender, and enzyme immunoassay (EIA) value distribution in rural Wyoming communities, including the median and range of optical densities (OD) for EIA readings

Characteristics of populations	Afton (N=253)	Alpine (N=84)	Thayne (N=148)
Age (y) Mean (SD) Median (range)	56 (15) 56 (16–92)	55 (12) 56 (20–78)	61 (14) 63 (16–92)
Male: female	120:133	39:45	66:82
OD (EIA units) Least squares mean Median (range)	0.357 0.349 (0.192–1.115)	0.340 0.343 (0.185–0.550)	0.359 0.353 (0.230–0.643)
EIA, 80th percentile N+ (%) EIA, 90th percentile N+ (%)	69 (27.3) 38 (15.0)	11 (13.1) 5 (6.0)	31 (20.9) 16 (10.8)

## Discussion

These data suggest that rural residents have greater exposure to an antigen or antigens that produce antibodies to the *E. coli* O157 LPS antigen than do urban residents. However, we cannot state with certainty that the precipitating antigen was actually a pathogenic *E. coli* O157:H7. Because the O157 LPS antigen can be expressed by nonpathogenic *E. coli* (*27*), *Citrobacter freundii* (*28*), and *E. hermanii* (*29*), knowing the nature of the antigen during the immunizing event in the participants studied is not possible. Nonetheless, antibodies to the *E. coli* O157 LPS antigen plausibly represent exposure to pathogenic *E. coli* O157:H7, especially as examples exist of asymptomatic carriage of *E. coli* O157:H7 inducing an antibody response to O157 LPS (*1*,*30*). We believe, therefore, that this serologic reactivity likely represents actual exposure to pathogenic *E. coli* O157:H7. Also, our assay did not distinguish the classes of antibodies that were reactive in the EIA, so we cannot make estimates about the timing of the exposure based on class of antibody detected. However, IgA, IgG, and IgM antibodies to the O157 LPS are each ephemeral after natural symptomatic infections (*31*). Thus, the antibodies that we detected in this study quite likely represent recent exposure to the antigen.

The EIA levels were not proportional to cattle density per land area, a value that was similar in each of the three study sites. Thus, human exposure to *E. coli* O157:H7 cannot be attributed simply to cattle presence within counties. However, in rural counties, a higher proportion of residents might be involved in activities that bring them in contact with *E. coli* O157:H7, including animal contact. Our survey was not designed to measure such exposures within counties. Indeed, cattle-to-human spatial proximity in Ontario is a risk factor for infection in a novel application of livestock density indicators and disease incidence (*32*). Alternatively, rural residents might have a higher frequency of exposure to wild animals that carry *E. coli* O157:H7, such as deer (*33*), their excreta, or water that has been contaminated with their excreta. However, if a link between any animal source of *E. coli* O157:H7 and human exposure to this pathogen is to be further investigated, population distributions within counties, proximities of humans to animals and their excreta, and presence of *E. coli* O157:H7 in the environment will all need to be examined to determine the modes of contact.

Our data differ from results of previous studies in terms of statistical analysis and also because we included a gradient of population density. Specifically, our principal analysis did not require assignment of persons to seropositive or seronegative status categories. While some relation might exist between percentage of persons who are designated as having reactive or nonreactive status on the basis of cutoffs and the comparative distribution of serologic reactivities in populations, we believe that, when examining continuous variables, a test that compares values as continuous measurements (such as analysis of variance) provides more information and is less arbitrary than the assignment of categorical positives and negatives. We did, however, also examine proportions above two cutpoints, and the same trends were noted. For unknown reasons, we identified higher EIA scores in younger study participants, whereas in Ontario, IgG antibodies to the O157 LPS were highest in participants in their fifth decade of life (*15*). Comparing our age-related EIA scores to those reported recently from Wisconsin, where older children had higher seropositive rates, is not possible because the latter study focused on a child population and did not evaluate older Wisconsin residents (*16*).

We caution against interpreting our data to mean that rural populations are immune to, and thereby protected from, *E. coli* O157:H7 infections. Many study participants had antibody levels that were probably too low to confer protection, even if one assumes that antibodies to this antigen protect against infection. In this regard, an *E. coli* O157:H7 infection, which almost always induces a brisk and high-titer humoral immune response to the O157 LPS antigen, may not confer a permanently protective response, as evidenced by documentations of recurrent infections (*34*,*35*). However, in a recent *E. coli* O157:H7 outbreak in the Star Valley, a higher frequency of antibody levels to the O157 LPS among the resident population was proposed as the reason for a lower attack rate among residents than among visitors (*19*). We also urge against generalizing the trend observed in this study, which is derived from the analysis of only three populations, to all rural populations, without additional, more widespread, studies because the populations studied might not be representative. Nonetheless, our data are consistent with the hypothesis that rural residence carries with it a greater risk for exposure to *E. coli* O157:H7 than does urban residence.

In summary, we have identified an age-dependent, gender-independent, risk for probable exposure to *E. coli* O157:H7 in persons living in rural communities. This exposure frequency is plausibly environmental, rather than foodborne, in origin because food is distributed widely throughout North America. However, the possibility exists that particular food consumption practices, such as drinking raw milk in rural communities, as has been noted in the United Kingdom (*36*,*37*), might have been responsible for this exposure. We also cannot exclude the possibility that the differences observed relate to the nature of the participants studied. That is, donors to blood banks might have different exposures than participants in lipid screenings at health fairs. Future studies should attempt to identify the points of exposure to this antigen, confirm that *E. coli* O157:H7 is, indeed, the source of the inciting antigen, and, if it is, minimize human contact with this pathogen.
